# Prevalence and Awareness of Carpal Tunnel Syndrome Among Adults in Tabuk City of Saudi Arabia: A Cross-Sectional Study

**DOI:** 10.7759/cureus.54076

**Published:** 2024-02-12

**Authors:** Hyder Mirghani, Areej A Aljohani, Abdulmajeed S Alharbi, Bandar S Alatawi, Fahad G Alanazi, Meshary D Alzahrani, Abdulaziz S AlJohani, Yasir M Alhusayni, Hassan A Alhwiti

**Affiliations:** 1 Internal Medicine, University of Tabuk, Tabuk, SAU; 2 Faculty of Medicine, University of Tabuk, Tabuk, SAU

**Keywords:** median nerve, peripheral entrapment neuropathy, saudi arabia, awareness, risk factors, prevalence, carpal tunnel syndrome

## Abstract

Background

Carpal Tunnel Syndrome (CTS) is a condition when the median nerve is entrapped and compressed within the wrist. It significantly affects the quality of life and work productivity of the affected individuals.

Aim

This study aimed to assess the prevalence of CTS and the risk factors associated with this condition among the general population in Tabuk City, Saudi Arabia, and to explore their knowledge of the causes, manifestations, and treatment options.

Methods

This cross-sectional study included male and female adult residents of Tabuk City aged 18 years and above who agreed to participate in the study. Data were collected using an online, self-administered questionnaire that was distributed to the public using different social media platforms.

Results

In this study, the prevalence of CTS was 3.4%. The presence of chronic diseases was a significant risk factor for the CTS (p = 0.003). Participants having chronic diseases were 6.370 times more likely to develop CTS (AOR: 6.370, 95% CI: 2.048 to 19.817). The participants had good levels of awareness about the causes (89.3%), clinical manifestations, and treatment of CTS (92.2%). There was a significant association between the level of knowledge about the causes of CTS and gender (p=0.014). Females (74.3%) showed a higher level of knowledge than males (25.7%). As well, the young (18-25) age group (67.9%) was more significantly aware of the causes of CTS in comparison to the other age groups (p=0.023).

Conclusion

The prevalence of carpal tunnel syndrome among the adult population in Tabuk City, Saudi Arabia, was 3.4%, and the significant underlying risk factors were chronic diseases such as diabetes mellitus, hypothyroidism, and rheumatoid arthritis. The level of awareness of CTS was satisfactory.

## Introduction

Carpal Tunnel Syndrome (CTS) constitutes the most common compression mononeuropathy in the upper extremities [1]. The condition is attributed to compression and traction of the short segment of the median nerve within the carpal tunnel. This might be complicated by ischemic injury of the nerve microcirculation. Consequently, a range of neurologic symptoms occurs along the palmar distribution of the median nerve in the hand [2,3].

Although most cases of CTS are idiopathic, certain risk factors have been identified, such as obesity, hormonal changes, pregnancy, trauma, wrist injuries, repetitive hand movements, and certain medical conditions such as diabetes mellitus, hypothyroidism, and rheumatoid arthritis. Aging, female gender, and genetic predisposition have also been implicated [4,5].

Diagnosis can be made clinically by a typical history of pain, numbness, and tingling as well as sensation loss along the thumb, index, middle finger, and radial side of the ring finger [6]. With further progression of the disorder, physical examination shows atrophy of the thenar muscle, weakness of the hand grip, and decreased fine motor coordination. Electrodiagnostic tests assist in confirming the diagnosis if uncertainty is present [7].

Carpal tunnel syndrome can affect the daily lives and work productivity of the affected individuals, with significant economic implications. Awareness about CTS helps early diagnosis which is crucial to preventing further nerve damage and to initiate appropriate treatment [8]. Globally, the prevalence of CTS among the general population is about 4 to 5% [5]. In Saudi Arabia, the exact prevalence of CTS is unclear. Few studies are concerned with the prevalence and awareness regarding CTS. Alyousef et al. [9] demonstrated a prevalence of 14% in Al-Majmaah City with sufficient awareness of the general population regarding the disorder, while Tawakul et al. [10] reported a lesser prevalence of 2% in the western region, with insufficient awareness.

Currently, there is no reported data about the prevalence or awareness regarding CTS among citizens of Tabuk City. Therefore, this study aimed to assess the prevalence and risk factors associated with CTS among the general population in Tabuk City, Saudi Arabia. Further, to explore their knowledge of the causes, manifestations, and treatment options.

## Materials and methods

Study design and settings

This cross-sectional observational study was conducted at the University of Tabuk, Saudi Arabia, between October and November 2023. Tabuk City is the capital of the Tabuk Region in North-western Saudi Arabia. Tabuk City is a rapidly growing urban center known for its diverse population, with a mix of urban and suburban areas and a wide range of socio-economic statuses.

Ethical considerations

This study obtained ethical approval from the Research Ethics Committee of the Faculty of Medicine, University of Tabuk, Saudi Arabia. We obtained informed consent from each participant before collecting the data. We informed them that their participation was voluntary, they had the right to withdraw anytime without completing the questionnaire, and that their responses would be anonymous and confidential. We did not offer any incentives for participation.

Sample size and sampling technique

The sample size was estimated with an online sample size calculator (Raosoft, http://www.raosoft.com/samplesize.html) using a margin of error of 5%, a confidence interval of 95%, assuming an average response for most of the questions of 50%, and depending on an average total population size in Tabuk City of 623,665. The minimum required sample size was 384 participants. We enrolled the participants using the non-probability conventional sampling technique.

Eligibility criteria

The study included both male and female adult residents of Tabuk City aged 18 years and above. Individuals under the age of 18, non-residents of Tabuk City, who declined to participate in the study, or who were unable to understand the study's purpose and provide informed consent (e.g., those with cognitive impairments) were excluded.

Data collection

We collected data using an online, self-administered questionnaire. This electronic survey was created using Google Forms and was distributed to the public using different social media platforms. The questionnaire was developed based on a review of the previously published research articles on carpal tunnel syndrome, and it was reviewed by experts in the field to ensure the clarity and validity of the questions [9,10]. Furthermore, the diagnosis of CTS was based on the American Academy of Orthopaedic Surgeons Guidelines [11]. The researchers translated the questionnaire to Arabic and revised it, and it was pre-tested on a small sample to refine any ambiguous or problematic items. The questionnaire included three parts. The first part was about the sociodemographic data and included questions about age, gender, nationality, marital status, education level, and occupation, besides inquiries about their weight and height. The second part included questions regarding a previous diagnosis of carpal tunnel syndrome by a physician and the presence of one or more chronic diseases. The third part assessed the awareness of the participants about carpal tunnel syndrome and included queries about the causes, symptoms, and potential treatment of carpal tunnel syndrome.

Statistical analysis

All data were tabulated and analyzed using the statistical package for the social sciences software program, SPSS Statistics for Windows, version 26 (IBM Corp., Armonk, NY). Awareness regarding causes, clinical manifestations, and potential treatments was classified into either “good” if the participants chose “yes” in at least one question or “poor” if they chose “no” in all questions. We presented qualitative data as frequencies and percentages and analyzed the possible associations between the sociodemographic variables and the prevalence as well as the awareness about CTS using Pearson’s Chi-square or Fisher Exact tests as appropriate. A multivariable forward stepwise logistic regression analysis was performed to identify the risk factors of carpal tunnel syndrome. The variables that showed a p-value ≤ 0.1 in the univariate analysis were entered as candidate covariates in the multivariable analysis. Results were shown as adjusted odds ratio and confidence intervals. A p-value <0.05 was considered statistically significant.

## Results

This study included 384 adults residing in Tabuk City. Most (72.4%) of them were Saudi females (97.7% and 72.4%, respectively) and almost two-thirds (65.9%) were aged 18-25 years. Single individuals constituted 70.8% while 27.9% were married. More than two-thirds (69.3%) joined the university, and 225 (58.6%) were students. Only 51 (13.3%) were obese, having a BMI ≥ 30 kg/m^2^ (Table 1).

**Table 1:** Sociodemographic characteristics of the study participants (N=384) N: number, BMI: body mass index

Sociodemographic characteristics		N=384	%
Gender	Female	278	72.4%
	Male	106	27.6%
Age groups	18 - 25	253	65.9%
	26 - 35	63	16.4%
	36 - 45	54	14.1%
	64 - 55	14	3.6%
Nationality	Saudi	375	97.7%
	Non-Saudi	9	2.3%
Marital status	Single	272	70.8%
	Married	107	27.9%
	Widowed	3	0.8%
	Divorced	2	0.5%
Education level	Intermediate	3	0.8%
	Secondary	69	18.0%
	University	266	69.3%
	Postgraduate	46	12.0%
Occupation	Student	225	58.6%
	Office work	66	17.2%
	Professional	30	7.8%
	Unemployed	29	7.6%
	Housewife	22	5.7%
	Retired	12	3.1%
Obesity (BMI ≥ 30 kg/m2)	No	333	86.7%
	Yes	51	13.3%

**Figure 1:**
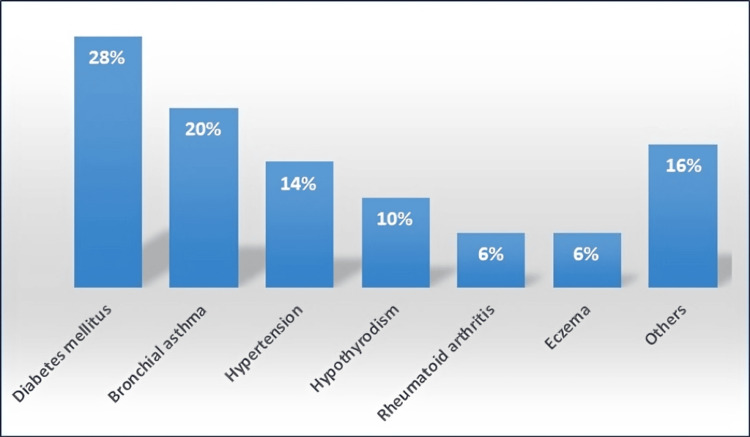
Frequency of chronic diseases among the study participants

Furthermore, 50 out of 384 (13%) participants reported having chronic diseases. Diabetes mellitus was the most frequent (28%), followed by bronchial asthma (20%), hypertension (14%), and hypothyroidism (10%) as illustrated in Figure 1.

**Figure 2:**
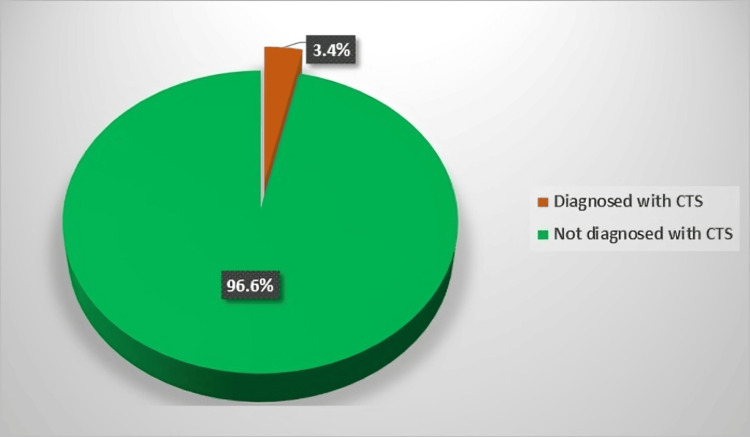
Prevalence of carpal tunnel syndrome (CTS) among the study participants

The prevalence of CTS diagnosis by a physician among the study participants was 3.4% (13/384) (Figure 2). The possible associations between CTS and some sociodemographic and medical factors were analyzed. There was a significant association between the presence of chronic diseases and having CTS (p<0.001). Six (46.2%) out of the 13 subjects diagnosed with CTS had chronic diseases. The presence of diabetes mellitus alone did not show a significant relationship with CTS (p=0.077). On the other hand, there were no significant associations between gender, age, practicing office or professional work, or obesity and the CTS (all p values >0.05) (Table 2).

**Table 2:** Factors associated with carpal tunnel syndrome *Significant at p<0.05

		Previous diagnosis with carpal tunnel syndrome				
		No		Yes		
		N	%	N	%	P-Value
Gender	Female	270	72.8%	8	61.5%	0.373
	Male	101	27.2%	5	38.5%	
Age groups	18 - 25	248	66.8%	5	38.5%	0.094
	26 - 35	59	15.9%	4	30.8%	
	36 - 45	50	13.5%	4	30.8%	
	64 - 55	14	3.8%	0	0.0%	
Office and professional work	No	281	75.7%	7	53.8%	0.099
	Yes	90	24.3%	6	46.2%	
Obesity	No	323	87.1%	10	76.9%	0.290
	Yes	48	12.9%	3	23.1%	
Diabetes mellitus	No	359	96.8%	11	84.6%	0.077
	Yes	12	3.2%	2	15.4%	

A multivariable forward stepwise logistic regression analysis revealed that the presence of chronic diseases was a significant risk factor for the CTS (p = 0.003). Participants with chronic diseases were 6.370 times more likely to develop CTS (AOR: 6.370, 95%CI: 2.048 to 19.817). Other candidate variables, including age, type of work (office or professional work), or the presence of diabetes mellitus alone were not significant risk factors (Table 3).

**Table 3:** A multivariable forward stepwise logistic regression model for identifying risk factors of carpal tunnel syndrome AOR: adjusted odds ratio, CI: confidence interval, *Significant at p<0.05.

Parameters	Beta coefficient	P-value	AOR	95% CI of AOR		Accuracy	P-Value
				Lower	Upper		
Chronic diseases	1.852	0.001*	6.370	2.048	19.817	96.6%	0.003*
Constant	-3.844	<0.001*					

**Figure 3:**
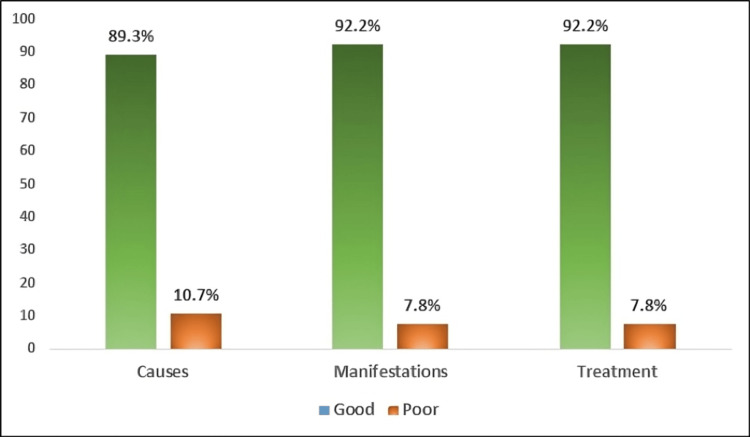
Levels of awareness about causes, clinical manifestations, and treatment of carpal tunnel syndrome.

Figure 3 shows that participants had good levels of awareness about the causes (89.3%), clinical manifestations, and treatment of CTS (92.2%).

Table 4 displays the knowledge of the study participants about CTS. Regarding the causes of CTS, 260 (67.7%) respondents recognized repeated physical activity. The second-most identified cause was trauma (55.7%), followed by arthritis (53.9%). Additionally, 204 (53.1%) and 159 (41.4%) participants identified wrist fractures/dislocations and tumors of the bone as potential causes of CTS. Inquiries about awareness of the clinical features of CTS revealed that wrist pain (73.7%), decreased overall hand grip (71.6%), and wasting in the hand (70.8%) were the most identified clinical manifestations of CTS. Additionally, 263 (68.5%) reported weakness of the thumb muscle, and 259 (67.4%) recognized tingling and numbness in the thumb, index, and middle fingers as clinical features of CTS. Furthermore, surgery and steroid injection were the most identified treatments for CTS (75.5% and 60.9%, respectively). Non-steroidal anti-inflammatory drugs were reported as potential treatment options by 222 (57.8%) respondents. Splinting (40.9%) was also identified as a potential treatment.

**Table 4:** Knowledge of the study participants about the causes, clinical symptoms, and potential treatments of carpal tunnel syndrome N: number

Parameters	N=384	%
Arthritis	207	53.9%
Trauma	214	55.7%
Bone tumor	159	41.4%
Multiple physical activities like using a computer and taping	260	67.7%
A wrist fracture or dislocation	204	53.1%
Pain in the wrist	283	73.7%
Tingling and numbness in the thumb, index, and middle fingers	259	67.4%
Decreased overall hand grip	275	71.6%
Weakness affecting the thumb muscle	263	68.5%
Wasting in the hand	272	70.8%
Oral analgesics	165	43.0%
Non-steroidal anti-inflammatory drugs	222	57.8%
Steroid injection	234	60.9%
Splint	157	40.9%
Surgery	290	75.5%

Table 5 demonstrates a significant association between the level of knowledge about the causes of CTS and gender (p=0.014). Females (74.3%) showed a higher level of knowledge than males (25.7%). As well, the young (18 - 25) age group (67.9%) was more significantly aware of the causes of CTS in comparison to the other age groups (p=0.023). Otherwise, there were no significant associations between the levels of awareness and the gender, age, education, or type of work of the participants.

**Table 5:** Associations between levels of awareness and some sociodemographic characteristics *Significant at p <0.05

		Causes				Clinical manifestations				Treatment			
		Good		Poor		Good		Poor		Good		Poor	
		N	%	N	%	N	%	N	%	N	%	N	%
Gender	Female	255	74.3	23	56.1	258	72.9	20	66.7	258	72.9	20	66.7
	Male	88	25.7	18	43.9	96	27.1	10	33.3	96	27.1	10	33.3
P-Value		0.014*				0.465				0.465			
Age groups	18 – 25	233	67.9	20	48.8	237	66.9	16	53.3	236	66.7	17	56.7
	26 – 35	53	15.5	10	24.4	58	16.4	5	16.7	57	16.1	6	20.0
	36 – 45	47	13.7	7	17.1	48	13.6	6	20.0	50	14.1	4	13.3
	64 – 55	10	2.9	4	9.8	11	3.1	3	10.0	11	3.1	3	10.0
P-Value		0.032*				0.158				0.213			
Education level	Intermediate	2	0.6	1	2.4	2	0.6	1	3.3	2	0.6	1	3.3
	Secondary	61	17.8	8	19.5	62	17.5	7	23.3	62	17.5	7	23.3
	University	239	69.7	27	65.9	247	69.8	19	63.3	247	69.8	19	63.3
	Postgraduate	41	12.0	5	12.2	43	12.1	3	10.0	43	12.1	3	10.0
P-Value		0.625				0.271				0.271			
	Student	207	60.3	18	43.9	211	59.6	14	46.7	212	59.9	13	43.3
	Office work	54	15.7	12	29.3	55	15.5	11	36.7	54	15.3	12	40.0
	Professional	27	7.9	3	7.3	29	8.2	1	3.3	28	7.9	2	6.7
	Housewife	19	5.5	3	7.3	20	5.6	2	6.7	21	5.9	1	3.3
	Unemployed	25	7.3	4	9.8	27	7.6	2	6.7	27	7.6	2	6.7
	Retired	11	3.2	1	2.4	12	3.4	0	0.0	12	3.4	0	0.0
P-Value		0.290				0.118				0.058			

## Discussion

Carpal tunnel syndrome is a musculoskeletal disorder caused mainly by strain and repetitive hand movement. The associated work absence and healthcare costs constitute a great socioeconomic burden. Adequate knowledge of the CTS is essential for early diagnosis and proper treatment [12]. The purpose of this study was to assess the prevalence, risk factors, and level of awareness and knowledge about CTS among the general population in Tabuk City, Saudi Arabia.

This study revealed a CTS prevalence of 3.4% (3/384) which was based on a previous diagnosis by a physician. The detected prevalence aligns with the reported global prevalence of CTS (4-5%) [5]. In comparison to previous studies in Saudi Arabia, the identified prevalence is higher than that reported by Tawakul et al. [10] in the Western region (2%), while it is much lower than the previously reported in Al-Majmaah City (14%) [9]. Furthermore, Altraifi et al. [13], in Hail City, reported a CTS prevalence of 24.1%. This high rate was based on the prevalence of self-reported symptoms by the participants as well as a previously confirmed diagnosis of CTS.

The observed differences in the prevalence of CTS among different regions of Saudi Arabia might be attributed to variances in the age and gender distribution among the studied population, besides work practices that require repetitive hand and wrist movements [14]. Genetic predisposition, which modifies the mechanical properties of tendons and other connective tissue structures within the carpal tunnel might also have a role [15].

Analysis of risk factors for CTS revealed a significant association between the presence of chronic diseases such as diabetes mellitus and hypothyroidism and having CTS. A significantly high percentage of individuals who developed CTS (46.2%) reported having chronic diseases. Logistic regression analysis documented a significant contribution of chronic diseases to the development of CTS, with 6.370 times increased risk of developing the disorder. However, the relationship between diabetes mellitus alone and developing CTS failed to reach a significant result. Most of the corresponding studies in Saudi Arabia [9,10,13] revealed similar findings. Alternatively, one study included a general population from different regions in Saudi Arabia that did not find an association between CTS and chronic disease despite the high frequency of diabetes as a comorbidity [16]. An earlier study in Boston, United States of America demonstrated diabetes mellitus and untreated hypothyroidism as common non-occupational risk factors for CTS [17]. Another case-control study in China revealed a significant association between chronic diseases such as diabetes and hypothyroidism and showed diabetes as a significant predictor of moderate and severe CTS [18]. It has been shown that diabetes can cause local microcirculatory disorders in the median nerve, followed by chronic peripheral neuropathy [19]. Further, a study reported a higher tendency to develop CTS among diabetic patients (30%) compared to non-diabetics (14%) [20]. The relationship between other chronic diseases like rheumatoid arthritis and CTS has been demonstrated by Devi et al. [21].

The participants of this study showed sufficient levels of overall awareness of CTS. High percentages displayed good levels of awareness about the causes (89.3%), clinical manifestations, and potential treatments (92.2%), where they have a piece of knowledge about the causes, suggestive clinical manifestations, and potential treatment of the disease. Regarding the causes of CTS, more than two-thirds (67.7%) identified repeated physical activity, and more than half identified trauma (55.7%), arthritis (53.9%), and wrist fractures (53.1%). Moreover, the diagnostic features of CTS, including wrist pain (73.7%), decreased overall hand grip (71.6%), wasting in the hand (70.8%), and tingling and numbness in the thumb, index, and middle fingers were identified. Concerning treatment of CTS, high percentages recognized surgery (75.5%) and steroid injection (60.9%) as options for treatment.

The detected sufficient levels of awareness among the study participants reflect their easy access to information via different sources such as online tools. Maintenance and improvement of public awareness about CTS is essential. This requires the collaboration of healthcare providers with community leaders and organizations. Ensuring awareness of the general population helps early appropriate treatment, prevents further nerve damage, and thereby, better outcomes and quality of life [22].

Previous studies in different regions of Saudi Arabia revealed contradictory results. Tawakul et al. [10] reported that residents in the Western region had inadequate knowledge about the causes (52.5%), clinical features (54.4%), and treatment (56%) of CTS. Public awareness of CTS in the Al-Jouf region was predominantly poor (74.8%) [23]. Whereas Alyousef et al. [9] reported sufficient awareness in Al-Majmaah City, Saudi Arabia about causes, features, and treatment. Also, Albaker et al. [16] reported that Saudis from different regions in Saudi Arabia were aware of CTS clinical symptoms. Another study found poor awareness of both Indian and Malaysian dentists about the link between chronic diseases and CTS, possible treatment by steroid injection, and preventive measures [21].

The present study also revealed that females (74.3%) were significantly more knowledgeable about the causes of CTS than males (74.3%). Also, the young (18-25 years) age group (67.9%) was more significantly aware of the causes of CTS in comparison to the other age groups. Otherwise, no significant relationships were found between the levels of awareness and the gender, age, education, or type of participants’ work. Similar findings have been reported by previous studies in Saudi Arabia [9,10,16,23]. Furthermore, Raman et al. found a significant association between the level of awareness of CTS and female gender in Kuwait [24].

## Conclusions

The findings of the present study disclosed a 3.4% prevalence of carpal tunnel syndrome among the adult population in Tabuk City, Saudi Arabia. Chronic diseases such as diabetes mellitus, hypothyroidism, and rheumatoid arthritis contributed significantly to the development of CTS; also the female gender shows a higher risk for developing CTS. Furthermore, there was a sufficient level of awareness of the causes, clinical features, and treatment of CTS. Still, there is a continuous need for awareness-raising campaigns about CTS and its symptoms. This will help people seek health care early, with better outcomes and quality of life.

Nevertheless, the reliance on a self-administered questionnaire, which is more prone to subjectivity, the dominance of females with a University degree, and the study being conducted in a single city limited our results. therefore, the current data cannot be generalized to the whole country.
